# Atrial Myxoma Presenting with Palpitations: A Case Report

**DOI:** 10.7759/cureus.4093

**Published:** 2019-02-19

**Authors:** George S Prousi, Joseph V Moran, Ross G Biggs

**Affiliations:** 1 Internal Medicine, Lehigh Valley Health Network, Allentown, USA; 2 Cardiology, Lehigh Valley Health Network, Allentown, USA

**Keywords:** myxoma, palpitations, transthoracic echocardiography, angiography, mitral valve, atrial septal defect

## Abstract

Atrial myxomas are a rare phenomenon and although benign, primary neoplasms of the heart can be burdensome depending on their location. Clinical symptoms are caused through a variety of mechanisms including conduction disturbances, obstruction, and valvular interference. Size and symptom development are strongly correlated and can almost always be detected by the use of echocardiography, magnetic resonance imaging or computed tomography. This is a case of a 62-year-old female with no significant past medical history presented to our facility with complaints of palpitations and associated dizziness for three months.

## Introduction

Atrial myxomas are the most common benign primary neoplasm of the heart, accounting for close to 80% of all cardiac tumors [1]. Although quite rare, cardiac tumors in general can present with a multitude of symptoms that are closely correlated with their location [2]. When symptoms are present, echocardiography and other imaging modalities almost always detect a lesion [3]. When found in the left atria, manifestations include conduction disturbances, obstruction, and valvular interference owing to symptoms including dyspnea, orthopnea, cough, edema and fatigue. Given that clinical symptoms overlap with congestive heart failure and other cardiac abnormalities, accurate diagnosis is imperative for appropriate treatment and prognosis.

## Case presentation

A 62-year-old female with no significant past medical history presented to our facility with complaints of palpitations and associated dizziness for three months. Prior work-up of her palpitations with Holter monitoring showed no irregularities. On arrival, she was in no acute distress and her palpitations had subsided. Vitals that were obtained were largely unremarkable except for a blood pressure of 142/77. Her EKG showed no acute irregularity and laboratory testing was within normal limits. On physical, a regular rate was observed, no murmurs, gallops or rubs were auscultated. She did not exhibit jugular venous distention or peripheral edema and other organ systems did not yield and irregularities. 

**Figure 1:**
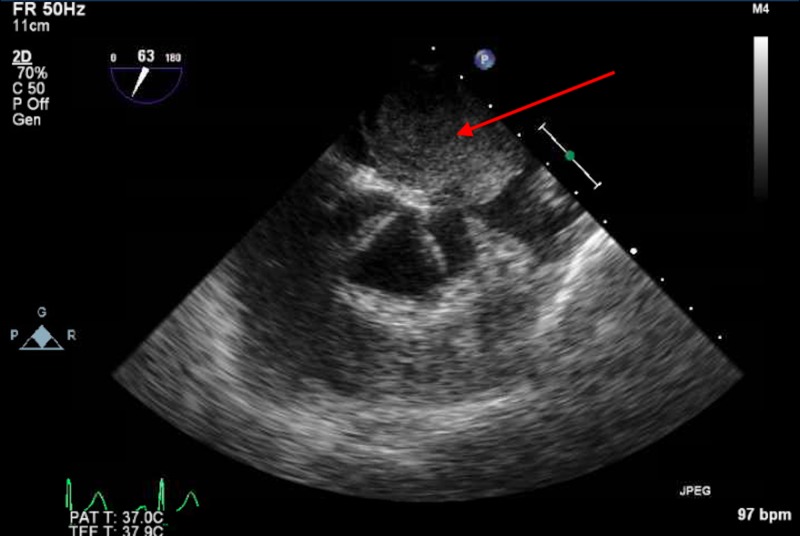
Transesophageal echocardiographic image showing left atrial mass (red arrow) seated above the aortic valve

**Figure 2:**
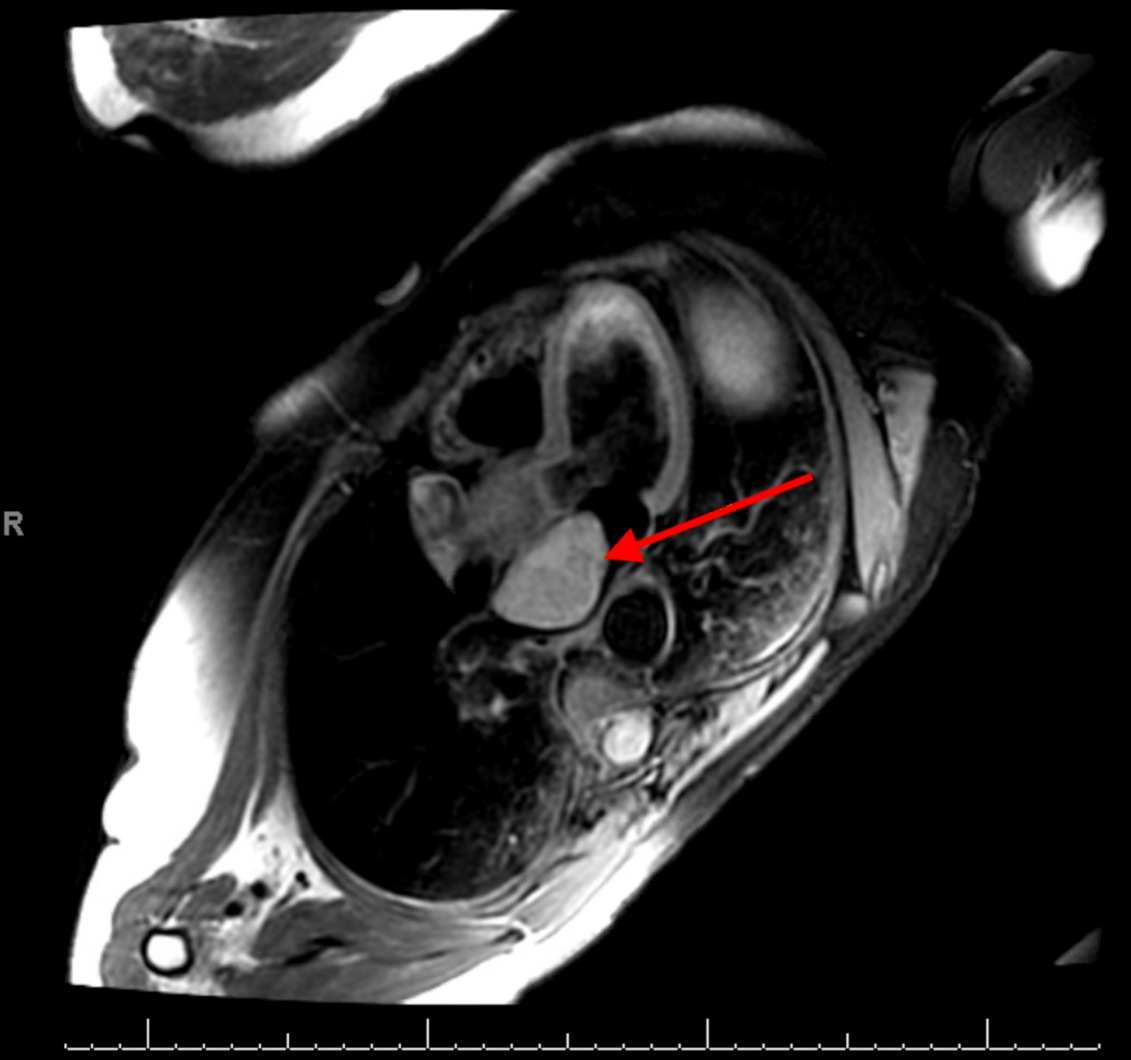
Cardiac magnetic resonance image depicting left atrial mass (red arrow)

The patient was admitted for further evaluation and a transthoracic echo was performed, revealing a 4.4 x 3.0-cm mass in the left atrium attached at the interatrial septum and aortomitral intervalvular fibrosa. Additional imaging studies including cardiac magnetic resonance imaging (MRI) and transesophageal echocardiography (TEE) were obtained for further confirmation of the mass and its location (Figures 1, 2). Surgical resection was planned, and pre-operative cardiac catheterization was performed which revealed mild prolapse of the mass causing intermittent obstruction of the mitral valve. 

The patient underwent full-thickness resection of the mass, resulting in an iatrogenic atrial septal defect which was closed with a Dacron patch. Her postoperative course was complicated by the development of intermittent complete heart block with junctional bradycardia and subsequent atrial fibrillation. She was evaluated by electrophysiology and a permanent pacemaker was implanted without complication. Discharge follow-up several weeks later noted the resolution of her prior symptoms, and repeat imaging showed no evidence of mass recurrence or mitral valve prolapse. 

## Discussion

Tumors of the heart commonly present with symptoms that correlate with the location, the degree of obstruction, and interference with heart valves or circulation. Clinical symptoms demonstrated in the literature include chest pain, dyspnea, and syncope but infrequently present with palpitations for an extended period of time [4-7]. Our patient had no past medical history and prior workup from an electrophysiological standpoint was within normal limits including EKG analysis and Holter monitoring. With the persistence of symptoms, echocardiography was obtained demonstrating a left atrial mass with further analysis showing intermittent mitral valve obstruction and resultant regurgitation. At present, evaluation of cardiac tumors aimed to determine size and location which is pertinent for further evaluation and eventual management. Given its wide availability, echocardiography continues to be the mainstay of the initial evaluation, whereas CT and MRI are used to provide additional details by offering insight as to the type of tumor [8-10]. 

When the diagnosis is confirmed, prompt resection is suggested to avoid the possibility of further manifestations including embolization or sudden cardiac death [11-12]. Outcomes are largely favorable with a mortality rate under 5% [11-13]; however, post-operative development of arrhythmias and atrioventricular conduction abnormalities have been reported in approximately 25% of patients [12]. Recurrence is rare although possible and is strongly correlated with a family history of myxoma development [13].

## Conclusions

This patient presented in an atypical fashion which lacked commonly associated symptoms including chest pain, dyspnea on exertion, and syncope. Imaging modalities including echocardiography, CT, and cardiac MRI are considered equally efficacious for the diagnosis of atrial tumors as they provide the location within the heart. Additional studies including angiography and biopsy are commonly required for further evaluation when surgery is planned. Surgical intervention consists of tumor resection and despite a low mortality rate, complications including arrhythmias and atrioventricular conduction delays are possible.
